# Gel immersion endoscopic mucosal resection for superficial esophageal cancer in a patient with pharyngoesophageal stenosis

**DOI:** 10.1055/a-2877-1961

**Published:** 2026-06-16

**Authors:** Tomoyuki Gurita, Yuji Urabe, Takahiro Uda, Yuichi Hiyama, Hidehiko Takigawa, Toshio Kuwai, Shiro Oka

**Affiliations:** 1 Department of Gastroenterology 68272 Hiroshima University HospitalHiroshimaHiroshimaJapan; 2 Hiroshima University Hospital 68272 Gastrointestinal Endoscopy and MedicineHiroshimaJapan

**Video 1** Gel immersion endoscopic mucosal resection in superficial esophageal cancer.


Endoscopic submucosal dissection (ESD) is widely used for the treatment of superficial esophageal cancers in Japan, and endoscopic mucosal resection (EMR) is commonly reserved for small lesions. Conventional EMR techniques, including cap-assisted EMR or the two-channel method, require dedicated accessories. 1​ ​2​ In contrast, gel-immersion EMR (GI-EMR) can be performed without distal attachment; the procedure has demonstrated favorable efficacy and safety in the treatment of gastric and duodenal tumors. 3​ ​4​ However, its use for superficial esophageal cancer, particularly in patients with pharyngoesophageal stenosis, has not been reported.

A 71-year-old man with a history of chemoradiotherapy (CRT) for pharyngeal cancer and ESD for esophageal cancer underwent follow-up endoscopy, which revealed a 10-mm type 0-IIa lesion in the mid-thoracic esophagus, considered to be limited to the epithelium and lamina propria mucosa and deemed suitable for endoscopic resection.

ESD was attempted but discontinued because the endoscope with a distal attachment could not be advanced due to post-CRT pharyngeal stenosis. Therefore, GI-EMR was performed ( **Video 1** ). An EG-840TP endoscope (Fujifilm, Tokyo, Japan) with an outer diameter of 7.9 mm—the largest endoscope that can pass through the stricture—was used.

**Fig. 1:**
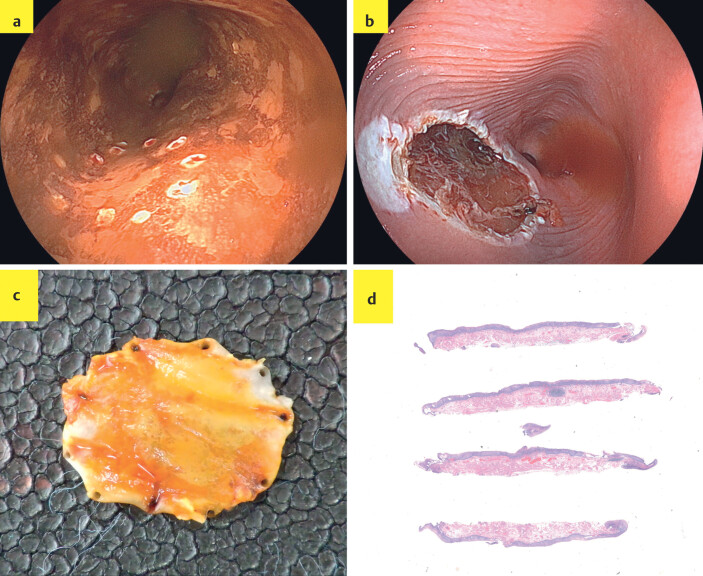
Gel immersion endoscopic mucosal resection in superficial esophageal cancer. ( **a** ) Superficial esophageal cancer following iodine staining and marking. ( **b** ) Post-resection ulcer base. ( **c** ) A resected specimen. ( **d** ) Histopathological findings showing squamous cell carcinoma confined to the mucosa, with negative margins.

After marking ( Fig. 1a ), gel injection created a clear working space. Following submucosal injection, en bloc resection was performed using a snare ( Fig. 1b and c ). After resection, minor bleeding was noted and successfully controlled using Coagrasper (Olympus, Tokyo, Japan). The total procedure time from marking to hemostasis was 18 minutes. No adverse events were reported. Histopathology showed squamous cell carcinoma confined to the mucosa (pT1a), without lymphovascular invasion and with negative margins, indicative of curative resection ( Fig. 1d ).

This case demonstrates that GI-EMR may be a feasible alternative when a standard-diameter endoscope cannot be advanced and ESD or cap-assisted EMR is technically challenging.

Endoscopy_UCTN_Code_TTT_1AO_2AG_3AC
